# Supplementary material for: Residency, movement patterns, behavior and demographics of reef manta rays in Komodo National Park
Source: PeerJ. 2022 May 16;10:e13302. doi: 10.7717/peerj.13302 (PMC9119296; doi:10.7717/peerj.13302)
Supplement: Supplemental Information 1 — The results for (A) male and (B) female manta rays at Karang Makassar (KM), Mawan (MW), and Manta Alley (MA), Komodo National Park. The records are from January 2013 to April 2018. [file peerj-10-13302-s001.docx]

| Model | Model description |
| --- | --- |
| A | Closed (1/*a*1 = *N)* |
| B | Closed (*a*1 = *N*) |
| C | Emigration/mortality  (a1 = emigration rate; 1/a2=*N*) |
| D | Emigration/mortality  (a1 = *N*; a2=mean residence) |
| E | Closed: emigration + re-immigration  (a1 = emigration rate;  a2/(a2 + a3) = proportion of population in study area at any time) |
| F | Emigration + re-immigration  (a1 = *N*; a2 = res time in; a3 = res time out) |
| G | Emigration + re-immigration + mortality |
| H | Emigration + re-immigration + mortality  (a1 = *N*; a2 = res time in; a3 = res time out; a4 = mort) |

|  | A) Males (*n* = 507) | |  | B) Females (*n* = 498) | | |
| --- | --- | --- | --- | --- | --- | --- |
| Location | KM | MW | MA | KM | MW | MA |
| Model | ΔQAIC | ΔQAIC | ΔQAIC | ΔQAIC | ΔAIC | ΔQAIC |
| A | 21508.8767 | 7201.8311 | 54.9819 | 78.1299 | 2.7558 | 40.8905 |
| B | 16.2036 | 7.7572 | 54.9818 | 78.1298 | 2.7558 | 40.8905 |
| C | 5.2733 | 5.1817 | 4.3354 | 6.4744 | ~0 | 23.6264 |
| D | 5.2733 | 5.1815 | 4.3349 | 6.4743 | 0 | 23.6261 |
| E | 8.8397 | 0.9351 | 2.4273 | 63.3293 | 3.9765 | 25.3614 |
| F | 1.2962 | 0.9178 | 2.4273 | 8.4743 | 3.9711 | 0 |
| G | 0 | 232.0128 | 1.514 | 0.3141 | 2.2387 | 496.5678 |
| H | ~0 | 0 | 0 | 0 | 3.0742 | 0.0272 |

*N* = population
