# Supplementary material for: Residency, movement patterns, behavior and demographics of reef manta rays in Komodo National Park
Source: PeerJ. 2022 May 16;10:e13302. doi: 10.7717/peerj.13302 (PMC9119296; doi:10.7717/peerj.13302)
Supplement: Supplemental Information 2 — The records are from January 2013 to April 2018. [file peerj-10-13302-s002.docx]

| Site | KM | MW | MA |
| --- | --- | --- | --- |
| Res time in (days) | 1.3 ± 5.4 | 0.8 ± 0.3 | 1.6 ± 12.9 |
| Res time out (days) | 6.9 ± 5.3 | 5.8 ± 4.4 | 5.8 ± 16.7 |
